# Solving Geophysical Inversion Problems with Intractable Likelihoods: Linearized Gaussian Approximations Versus the Correlated Pseudo-marginal Method

**DOI:** 10.1007/s11004-023-10064-y

**Published:** 2023-06-02

**Authors:** Lea Friedli, Niklas Linde

**Affiliations:** Institute of Earth Sciences, University of Lausanne, Lausanne, Switzerland

**Keywords:** Inverse theory, Intractable likelihood, Latent variable model, Hydrogeophysics

## Abstract

A geophysical Bayesian inversion problem may target the posterior distribution of geological or hydrogeological parameters given geophysical data. To account for the scatter in the petrophysical relationship linking the target parameters to the geophysical properties, this study treats the intermediate geophysical properties as latent (unobservable) variables. To perform inversion in such a latent variable model, the intractable likelihood function of the (hydro)geological parameters given the geophysical data needs to be estimated. This can be achieved by approximation with a Gaussian probability density function based on local linearization of the geophysical forward operator, thereby, accounting for the noise in the petrophysical relationship by a corresponding addition to the data covariance matrix. The new approximate method is compared against the general correlated pseudo-marginal method, which estimates the likelihood by Monte Carlo averaging over samples of the latent variable. First, the performances of the two methods are tested on a synthetic test example, in which a multivariate Gaussian porosity field is inferred using crosshole ground-penetrating radar first-arrival travel times. For this example with rather small petrophysical uncertainty, the two methods provide near-identical estimates, while an inversion that ignores petrophysical uncertainty leads to biased estimates. The results of a sensitivity analysis are then used to suggest that the linearized Gaussian approach, while attractive due to its relative computational speed, suffers from a decreasing accuracy with increasing scatter in the petrophysical relationship. The computationally more expensive correlated pseudo-marginal method performs very well even for settings with high petrophysical uncertainty.

## Introduction

This work targets a Bayesian inverse problem in which the posterior distribution of target geological or hydrogeological parameters $\varvec{\theta }$ are inferred from geophysical data ${\textbf{y}}$. Petrophysical relationships linking (hydro)geological variables (e.g., permeability, clay fraction, salinity) to geophysical properties (e.g., dielectric permittivity, electrical conductivity, magnetic susceptibility) must then be introduced (e.g., Hinnell et al. 2010; Kowalsky et al. 2005). Such relationships are often inherently uncertain (e.g., Mavko et al. 2009), however, in most hydrogeophysical inversion studies targeting hydrogeological properties, the predictive power of the petrophysical relationship is assumed to be perfect provided that the right parameter values are used (e.g., Lochbühler et al. 2014; Kowalsky et al. 2005). Brunetti and Linde (2017) show that this assumption may lead to bias, too narrow uncertainty bounds and overly variable parameter estimates.

Brunetti and Linde (2017) distinguish three sources of uncertainty in the petrophysical relationship: model, parameter and prediction uncertainty. While the first two result from uncertainty related to the choice of the petrophysical model and its parameter values, the latter arises from scatter and bias around the calibrated model. As in Brunetti and Linde (2017), only petrophysical prediction uncertainty is considered here, using a latent variable model formulation which expresses the geophysical properties as ${\textbf{X}} = {\mathcal {F}}(\varvec{\theta } ) + \varvec{\varepsilon _{P}}$, with ${\mathcal {F}}(\cdot )$ being the petrophysical relationship and $\varvec{\varepsilon _{P}}$ the petrophysical prediction error (PPE). The inclusion of the random effect of the PPE in the latent variable ${\textbf{X}}$ makes the likelihood function $p({\textbf{y}} |\varvec{\theta } )$ intractable. In this study, two alternative methods are investigated to approximate this likelihood function in a Metropolis–Hastings algorithm (MH algorithm; Metropolis et al. 1953; Hastings 1970). The first approach is a Gaussian approximation based on local linearization of the geophysical forward operator. Thereby, the effect of the noise in the petrophysical relationship is included by a corresponding addition to the data covariance matrix. This new approach which is similar to the so-called delta method (Van der Vaart 2000) was suggested by Linde et al. (2017), but it has remained untested to date. This approximate method is compared against the correlated pseudo-marginal (CPM) method of Deligiannidis et al. (2018), which is based on the pseudo-marginal (Beaumont 2003; Andrieu and Roberts 2009,PM) method using Monte Carlo sampling of the latent variable to estimate the likelihood. Friedli et al. (2022) introduced and adapted the CPM method to a geophysical setting and demonstrated that in data-rich geophysical settings with low noise levels, it is essential to both use a well-working importance sampling strategy for the draws of latent variables and to correlate the latent samples used in the proposed and current states of the Markov chain.

In Friedli et al. (2022), the CPM method is compared to the original formulation of lithological tomography (Bosch 1999) and the so-called full inversion approach of Brunetti and Linde (2017). This latter method avoids intractable likelihood functions by targeting the joint posterior PDF $(\varvec{\theta } , {\textbf{x}} ) \mapsto p(\varvec{\theta } , {\textbf{x}} | {\textbf{y}} )$ of the hydrogeological and geophysical parameters. Within the original lithological tomography method, first the target variable is sampled using the proposal scheme of the MH and second, one realization of the latent variable is drawn with conditional sampling. This actually represents a simplified form of the PM method with only one latent variable sample and without importance sampling. The original form of lithological tomography leads to high variability in the estimate of the likelihood function, with the consequence of the algorithm often being highly inefficient (Brunetti and Linde 2017). Within the full inversion of Brunetti and Linde (2017), the latent variables are treated as additional target variables and an MH proposal scheme is used to draw new realizations of both. Friedli et al. (2022) show that this approach becomes inefficient with increasing dimensionality of the target and latent space and suffers from strong (posterior) correlations between the target and latent variables. Friedli et al. (2022) present a comparison of CPM with the original lithological tomography and full inversion approaches in a weakly non-linear setting showing that the CPM method outperforms the others by greatly enhancing the posterior exploration. While the CPM method already has been tested for geophysical inversion problems, the linearized Gaussian approach has not been applied to far and in this present study, the focus is on comparing this approximate approach against the general CPM method.

As a synthetic test case, a similar setting as in Friedli et al. (2022) is considered and multi-Gaussian porosity fields are inferred using crosshole ground-penetrating radar (GPR) first-arrival travel times. As in Friedli et al. (2022), a high-dimensional parameterization of the target porosity field is used. Subsequently, a sensitivity analysis is made to explore the performances of the linearized Gaussian approach and the CPM method as a function of increasing petrophysical prediction uncertainty. To avoid the challenges of a very-high dimensional target space in this sensitivity analysis, the complexity of the porosity field is reduced and it is assumed to be layered.

This contribution is a natural extension of the study by Friedli et al. (2022), which only considered the CPM method. While the fundamental concepts of the considered problem and the CPM method are repeated, the introduction and assessment of the linearized Gaussian approach is completely new. The manuscript is structured as follows: Sect. 2 gives a methodological overview of the considered latent variable model, Bayesian inference with intractable likelihoods, the linearized Gaussian approximation approach, the CPM method and the performance assessment metrics. Section 3 presents the results of our synthetic case study inferring multi-Gaussian porosity fields. In Sect. 4, the sensitivity analysis is presented. Finally, the study finishes with a discussion and conclusions in Sects. 5 and 6.

## Methodology

### Latent Variable Model

In the considered setting, the data vector ${\textbf{y}} =(y_1, y_2,\ldots ,y_T)$ (geophysical data) is given by,

1 $$\begin{aligned} {\textbf{Y}} = {\mathcal {G}}({\textbf{X}} ) + {{\varvec{\varepsilon }}_{{\varvec{O}}}}= {\mathcal {G}}({\mathcal {F}}(\varvec{\theta } ) + \varvec{\varepsilon _{P}}) + {{\varvec{\varepsilon }}_{{\varvec{O}}}}, \end{aligned}$$

with ${\textbf{X}} =(X_1,X_2,\ldots ,X_L)$ denoting the latent variable (geophysical property) and $\varvec{\theta } =(\theta _1, \theta _2,\ldots \theta _d)$ the target parameters (hydrogeological parameters). The variable ${\textbf{X}}$ is referred to as a latent because it includes the PPE $\varvec{\varepsilon _{P}}$, which is unobservable (latent) but affects the observations. ${\mathcal {G}}: {\mathbb {R}}^L \rightarrow {\mathbb {R}}^T$ with ${{\varvec{\varepsilon }}_{{\varvec{O}}}}$ and ${\mathcal {F}}: {\mathbb {R}}^d \rightarrow {\mathbb {R}}^L$ with $\varvec{\varepsilon _{P}}$ refer to the physical forward solver with the observational noise and the petrophysical relationship with the PPE, respectively. In what follows, random variables and random vectors are referred to with upper-case letters and realizations thereof with lower-case letters. Assuming Gaussian errors, it holds,

2 $$\begin{aligned} p( {\textbf{x}} | \varvec{\theta } )= \varphi _{L}({\textbf{x}} ; {\mathcal {F}}(\varvec{\theta } ), \varvec{\Sigma _P}), \quad p({\textbf{y}} | \varvec{\theta } , {\textbf{x}} )=\varphi _{T}({\textbf{y}} ; {\mathcal {G}}({\textbf{x}} ), \varvec{\Sigma _{{\textbf{Y}} }}), \end{aligned}$$

with $\varphi _{M}(\cdot ; \varvec{\mu }, \varvec{\Sigma })$ denoting the PDF of a *M*-variate normal distribution with mean $\varvec{\mu }$ and covariance matrix $\varvec{\Sigma }$. In the test example, the target parameters $\varvec{\theta } =(\theta _1, \theta _2,\ldots \theta _d)$ describe a Gaussian random field parameterized on a grid of size $D \times D (d = D^2)$. It holds,

3 $$\begin{aligned} p(\varvec{\theta } ) = \varphi _{D^2}(\varvec{\theta } ; \varvec{\mu _{\theta }}, \varvec{\Sigma _{\theta }}), \end{aligned}$$

and it is assumed that the mean $\varvec{\mu _{\theta }}$ and the covariance matrix $\varvec{\Sigma _{\theta }}$ of the target field are known.

### Bayesian Inference and Intractable Likelihoods

Bayesian inversion problems target the posterior probability density function (PDF) $p(\varvec{\theta } | {\textbf{y}} )$ of the model parameters $\varvec{\theta }$ given the measurements ${\textbf{y}}$. In Bayes’ theorem, this posterior PDF is given by,

4 $$\begin{aligned} p(\varvec{\theta } | {\textbf{y}} ) = \frac{p(\varvec{\theta } ) p({\textbf{y}} | \varvec{\theta } ) }{p({\textbf{y}} )}, \end{aligned}$$

with the prior PDF $p(\varvec{\theta } )$ of the model parameters, the likelihood function $p({\textbf{y}} | \varvec{\theta } )$ and the evidence $p({\textbf{y}} )$. As it is not possible to sample directly from the posterior, we use the Metropolis–Hastings algorithm (MH algorithm; Metropolis et al. 1953; Hastings 1970). At iteration *j*, the MH algorithm proposes a new model realization using the model proposal density $q(\cdot | \varvec{\theta }^{(j-1)} )$, which is then accepted or rejected based on the acceptance probability,

5 $$\begin{aligned} \alpha _{MH} \left( \varvec{\theta }^{(j-1)} , \varvec{\theta }^{(j)} \right) = \min \biggl \{ 1, \frac{q(\varvec{\theta }^{(j-1)} | \varvec{\theta }^{(j)} )p(\varvec{\theta }^{(j)} )p({\textbf{y}} | \varvec{\theta }^{(j)} )}{q(\varvec{\theta }^{(j)} | \varvec{\theta }^{(j-1)} )p(\varvec{\theta }^{(j-1)} )p({\textbf{y}} |\varvec{\theta }^{(j-1)} )} \biggr \}. \end{aligned}$$

To implement the MH algorithm, the likelihood function $\varvec{\theta } \mapsto ~p({\textbf{y}} | \varvec{\theta } )$ has to be evaluated,

6 $$\begin{aligned} p({\textbf{y}} | \varvec{\theta } ) = \int p({\textbf{y}} | \varvec{\theta } , {\textbf{x}} ) p({\textbf{x}} | \varvec{\theta } ) \textrm{d} {\textbf{x}} . \end{aligned}$$

In a latent variable model, this integral has generally no analytical form, leading to an intractable likelihood function.

#### Proposal Scheme

When applying the MH algorithm to generate posterior samples, it is essential to choose a well-working proposal density $q(\cdot | \varvec{\theta }^{(j-1)} )$. Cotter et al. (2013) showed that standard random walk MCMC algorithms entail highly inefficient performance and strong dependence on the discretization when targeting high-dimensional Gaussian random fields. As a solution, they suggest proposal schemes that preserve the prior PDF, resulting in an MH algorithm for which the acceptance ratio only depends on the likelihoods. In geophysics, this proposal scheme is known as the extended Metropolis algorithm (Mosegaard and Tarantola 1995). If the target space is high-dimensional, the prior-preserving proposal scheme still needs to be chosen carefully (Ruggeri et al. 2015). Therefore, Friedli et al. (2022) introduce a prior-preserving version of the adaptive multi-chain algorithm DREAM_(ZS)_ (DiffeRential Evolution Adaptive Metropolis using an archive of past states; Laloy and Vrugt 2012).

### Gaussian Approximation of the Intractable Likelihood

The new approach to approximate the intractable likelihood (Eq. 6) relies on the linearized Gaussian approximation proposed by Linde et al. (2017). Here, the data covariance matrix given by the observational noise $\varvec{\Sigma }_{{\textbf{Y}} }$ (Eq. 2) is adjusted by adding an additional contribution accounting for petrophysical prediction uncertainty. For our latent variable model, a first-order Taylor expansion of ${\textbf{x}} \mapsto {\mathcal {G}}({\textbf{x}} )$ around ${\mathcal {F}}(\varvec{\theta } )$ is used,

7 $$\begin{aligned} {\textbf{Y}} = {\mathcal {G}}({\mathcal {F}}(\varvec{\theta } ) + \varvec{\varepsilon _{P}}) + {{\varvec{\varepsilon }}_{{\varvec{O}}}}\approx {\mathcal {G}}({\mathcal {F}}(\varvec{\theta } ) ) + {\textbf{J}}_{{\mathcal {F}}(\varvec{\theta } )} \varvec{\varepsilon _{P}}+ {{\varvec{\varepsilon }}_{{\varvec{O}}}}, \end{aligned}$$

with ${\textbf{J}}_{{\mathcal {F}}(\varvec{\theta } )}$ denoting the Jacobian (sensitivity) matrix of the forward solver corresponding to ${\mathcal {F}}(\varvec{\theta } )$. Due to its dependence on ${\mathcal {F}}(\varvec{\theta } )$, the sensitivity matrix is evolving between iterations (with changing $\varvec{\theta }$). Using Gaussian assumptions for $p({{\varvec{\varepsilon }}_{{\varvec{O}}}}) = \varphi _T({{\varvec{\varepsilon }}_{{\varvec{O}}}}; {\varvec{0}}, \varvec{\Sigma }_{{\textbf{Y}} })$ and $p(\varvec{\varepsilon _{P}}) = \varphi _L(\varvec{\varepsilon _{P}}; {\varvec{0}}, \varvec{\Sigma _{P}})$ (Eq. 2), the likelihood function (Eq. 6) is approximated by,

8 $$\begin{aligned} {\hat{p}}({\textbf{y}} | \varvec{\theta } )&= \varphi _T({\textbf{y}} ; \mu _{{\textbf{Y}} }, \overset{\sim }{\varvec{\Sigma }_{{\textbf{Y}} }}) \quad \text { with } \quad \mu _{{\textbf{Y}} } = {\mathcal {G}}({\mathcal {F}}(\varvec{\theta } )) \quad \text { and } \quad \overset{\sim }{\varvec{\Sigma }_{{\textbf{Y}} }} = {\textbf{J}}_{{\mathcal {F}}(\varvec{\theta } )}^T \varvec{\Sigma _{P}}{\textbf{J}}_{{\mathcal {F}}(\varvec{\theta } )} + \varvec{\Sigma }_{{\textbf{Y}} }. \end{aligned}$$

**Fig. 1:**
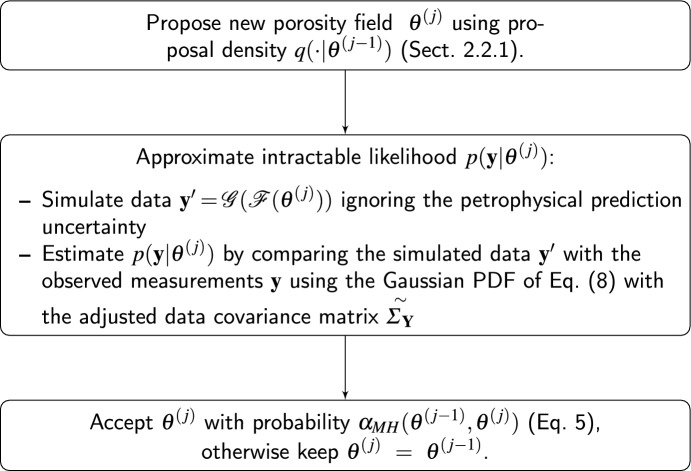
Flow chart illustrating the LinGau method at iteration *j*

As the linearization is made around $\mathcal {G}(\mathcal {F}(\varvec{\theta } ))$ and not around $\mathcal {G}(\mathcal {F}(\varvec{\theta } ) + \varvec{\varepsilon _{P}})$, errors arise when the resulting Jacobians differ. For a linear geophysical problem, there are no approximation errors. Figure 1 shows a flow chart describing this approach at iteration *j*; in what follows, this method will be referred to as LinGau.

### Correlated Pseudo-marginal Method

The pseudo-marginal method (Beaumont 2003; Andrieu and Roberts 2009) estimates the intractable likelihood (Eq. 6) by Monte Carlo averaging over samples of the latent variable,

9 $$\begin{aligned} {\hat{p}}_N({\textbf{y}} | \varvec{\theta } ) = \frac{1}{N} \sum \limits _{n=1}^{N} w({\textbf{y}} | {\textbf{X}}_n, \varvec{\theta } ), \quad \text {with} \quad w({\textbf{y}} | {\textbf{X}}_n, \varvec{\theta } ) = \frac{ p({\textbf{y}} | \varvec{\theta } , {\textbf{X}}_n) p({\textbf{X}}_n| \varvec{\theta } ) }{ m({\textbf{X}}_n | \varvec{\theta } ) }, \end{aligned}$$

where ${\textbf{X}}_n \overset{i.i.d}{\sim }\ m(\cdot | \varvec{\theta } )$ for $n=1,2,\ldots ,N$ with $m(\cdot | \varvec{\theta } )$ denoting an importance density function. This implies that after proposing a new target parameter $\varvec{\theta }$, different latent variable realizations ${\textbf{X}}_n$ with the same $\varvec{\theta }$ and different PPE $\varvec{\varepsilon _{P}}$ are sampled. Then, the likelihood of each realization can be calculated and the intractable likelihood function is estimated by averaging over the obtained values. To account for the influence of importance sampling on the draws of the latent variable, weighted averaging has to be applied. Using this non-negative unbiased estimator of the likelihood leads to a MH algorithm sampling the same posterior distribution as one using the true likelihood (Beaumont 2003).

To obtain an efficient algorithm, it is crucial that the variance of the log-likelihood ratio estimator used in each MH step is low enough (Doucet et al. 2015). This can be ensured by choosing a well-working importance sampling density and by selecting a number of latent variable samples *N* which is high enough. Following Friedli et al. (2022), a Gaussian approximation ${\textbf{x}} \mapsto \varphi _{L}({\textbf{x}} ; \varvec{\mu _{IS}},\varvec{\Sigma _{IS}})$ of ${\textbf{x}} \mapsto p({\textbf{x}} |\varvec{\theta } , {\textbf{y}} )$ is used as importance density, which implies the same linearization of the forward operator as in the LinGau approach (Eq. 7). An inappropriate linearization will lead to errors in the LinGau estimates, while it will only affect the efficiency of the pseudo-marginal method. To reduce the magnitude of *N*, Deligiannidis et al. (2018) introduced the correlated pseudo-marginal (CPM) method by which the samples of latent variables used in the likelihood ratio estimator are correlated. Assuming a standard-normal distributed latent variable ${\textbf{X}}$, the CPM method correlates one draw of iteration *j* with one of the former by,

10 $$\begin{aligned} {\textbf{X}}^{(j)}= & {} \rho {\textbf{X}}^{(j-1)} + \sqrt{1-\rho ^2} \varvec{\epsilon }, \text { with } \rho \in (-1,1) \text { and } \nonumber \\ \varvec{\epsilon }= & {} (\epsilon _1, \epsilon _2,\ldots ,\epsilon _L ), \epsilon _i \overset{i.i.d.}{\sim }\ {\mathcal {N}}(0,1). \end{aligned}$$

**Fig. 2:**
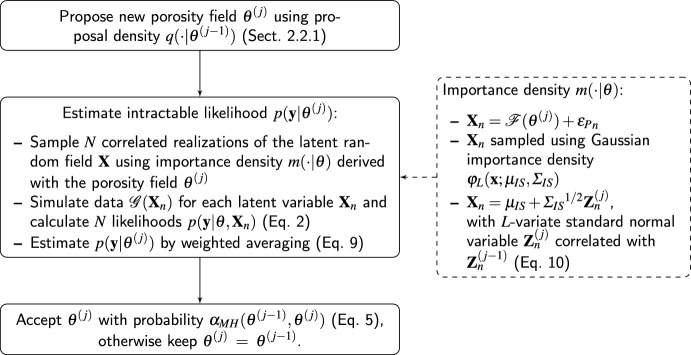
Flow chart illustrating the CPM method with importance sampling at iteration *j*

The general applicability of the CPM method is not limited by the assumption that the latent variable has a standard-normal distribution since numerous distributions can be obtained by transformations from standard normal variates (e.g. Chen et al. 2018). The procedure of the CPM method in iteration *j* is illustrated in Fig. 2. Further details about the method can be found in Friedli et al. (2022).

### Performance Assessment

The primarily focus of the assessment of the inversion results is on the exploration of the posterior PDF. To declare convergence of the MCMC chains, the ${\hat{R}}$-statistic of Gelman and Rubin (1992) is used. Subsequently, the posterior samples obtained with the considered methods are compared. For a numerical assessment of the posterior estimates, the logarithmic score (logS; Good 1952) is employed. This is a so-called scoring rule (Gneiting and Raftery 2007) evaluating the accuracy of a predictive PDF $\varvec{\theta } \mapsto {\hat{p}}(\varvec{\theta } )$ with respect to a true value $\varvec{\theta } _{\textrm{true}}$. The logarithmic score is defined as $\text {logS}({\hat{p}},\varvec{\theta } _{\textrm{true}}) = -\log {\hat{p}}(\varvec{\theta } _{\textrm{true}})$ and kernel density estimates with manually-selected bandwidths are applied to transform the posterior samples into a PDF. Furthermore, the number of target parameters in which the true porosity value $\theta _{\textrm{true}}$ is in the range of the posterior samples is considered, as well as the spread of the posterior samples as quantified by their standard deviations.

## Case Study

The new linearized Gaussian approach is compared against the CPM method using the first case study considered by Friedli et al. (2022).

### Synthetic Data Generation

**Fig. 3:**
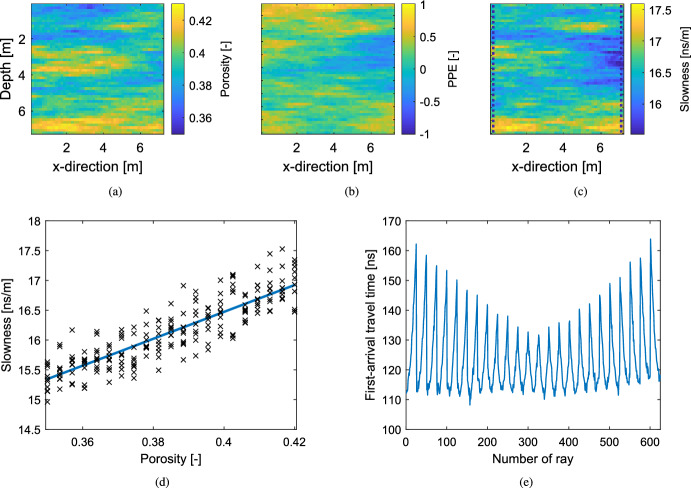
The synthetic “true” model adapted from Friedli et al. (2022): (**a**) porosity field $\theta _{\textrm{true}}$, (**b**) PPE field $\varvec{\varepsilon _{P}}_{\textrm{true}}$, (**c**) slowness field ${\textbf{x}} _{\textrm{true}}$, (**d**) scatter plot of porosity and slowness values per grid cell, the line depicts the petrophysical relationship (Eq. 11) without considering PPE and (**e**) set of noise-affected first-arrival travel times ${\textbf{y}}$

The considered model domain is a water-saturated subsurface area of 7.2 m $\times$ 7.2 m. As in Friedli et al. (2022), the target porosity field is assumed to be a Gaussian random field with known mean ($\varvec{\mu _{\theta }}=0.39$) and exponential covariance function. For the latter, a sill of $2 \times 10^{-4}$ is assumed such as geometric anisotropy with the main, horizontal direction having an integral scale of 5.4 m and the integral scale ratio between the horizontal and vertical direction being 0.13. For the parameterization of the porosity field, a regular (50 $\times$ 50)-dimensional grid ($D^2=2500$) is used. The “true” porosity field $\theta _{\textrm{true}}$ for this case study is depicted in Fig. 3a. Given the porosity $\varvec{\theta }$, the dielectric constant $\varvec{\kappa }$ is predicted using the complex refractive index model (CRIM; Roth et al. 1990), from which the slowness field (our latent variable ${\textbf{X}}$) can be derived,

11 $$\begin{aligned} {\textbf{x}} = \sqrt{c ^{-2}\varvec{\kappa }} + \varvec{\varepsilon _{P}}= \frac{1}{c} \big ( \sqrt{\kappa _s } + (\sqrt{\kappa _w }-\sqrt{\kappa _s }) \varvec{\theta } \big ) + \varvec{\varepsilon _{P}}, \end{aligned}$$

with $$\kappa _w $$ and $$\kappa _s $$ denoting the dielectric constants of water [81] and mineral grains [5], respectively, and $$c $$ referring to the speed of light in vacuum [0.3 m/ns]. A PPE $$\varvec{\varepsilon _{P}}$$ is added (Fig. 3b), which is a realization of a centred Gaussian random field over a regular two dimensional grid of size $$50 \times 50$$. Thereby, the same correlation structure as for the porosity field is used with a sill of $$7.11 \times 10^{-2}\,\textrm{ns}^2/\textrm{m}^2$$. The “true” slowness field is depicted in Fig. 3c while Fig. 3d shows a scatter plot depicting the influence of the PPE on the slowness values.

The 625 first-arrival travel times are generated using 25 equidistant GPR transmitters located on the left side and 25 receivers on the right side of the model domain (the transmitter-receiver layout is shown in Fig. 3c). As forward solver ${\textbf{y}} \mapsto {\mathcal {G}}({\textbf{y}} )$, the non-linear (eikonal) solver time2D of Podvin and Lecomte (1991) is used. The observational noise ${{\varvec{\varepsilon }}_{{\varvec{O}}}}$ is assumed to be *i*.*i*.*d*. centered normal with a standard deviation of 1 ns. The noise-affected synthetic first-arrival traveltimes are depicted in Fig. 3e.

### Inversion Setting and Prior Assumptions

As the considered target space is high-dimensional with 2500 unknown parameters, it is crucial to choose a well-working proposal scheme for the MH algorithm. Due to its convincing performance in Friedli et al. (2022), this study relies on prior-preserving DREAM_(ZS)_ proposals (Sect. 2.2.1) and four MH chains are run in parallel. For both the LinGau and the CPM method, the linearization used to adjust the data covariance matrix and the importance sampling, respectively, is updated every tenth MCMC iteration. For the CPM method, a configuration of $N=10$ and $\rho =0.95$ is used as this choice guarantees an appropriate variance of the log-likelihood ratio estimator (see, Friedli et al. 2022).

For the prior on porosity, a Gaussian PDF $p(\varvec{\theta } ) = \varphi _{2500}(\varvec{\theta } ; \varvec{\mu _{\theta }}, \varvec{\Sigma _{\theta }})$ with known mean $\varvec{\mu _{\theta }}$ and covariance structure $\varvec{\Sigma _{\theta }}$ (the same values as for the data generation) is assumed. Then, the 2500-dimensional vector ${\textbf{Z}}$ defining the porosity by $\varvec{\theta } = \varvec{\mu _{\theta }}+ \varvec{\Sigma _{\theta }}^{1/2} {\textbf{Z}}$ is inferred, with ${\textbf{Z}}$ having a multivariate standard-normal prior PDF. For the PPE $\varvec{\varepsilon _{P}}$ also a Gaussian prior PDF $p(\varvec{\varepsilon _{P}}) = \varphi _{2500}(\varvec{\theta } ; 0, \varvec{\Sigma _{P}})$ with known covariance structure $\varvec{\Sigma _{P}}$ is used. For the likelihood function, centred independent Gaussian measurement errors ${{\varvec{\varepsilon }}_{{\varvec{O}}}}$ with a standard deviation of 1 ns as in the data generation process are assumed.

The LinGau and CPM methods are compared with an inversion ignoring the petrophysical prediction uncertainty (No PPE). In this case, the intractable likelihood $p({\textbf{y}} | \varvec{\theta } )$ is estimated by $\varphi _T({\textbf{y}} ; {\mathcal {G}}({\mathcal {F}}(\varvec{\theta } )), \varvec{\Sigma }_{{\textbf{Y}} })$. As prior-preserving DREAM_(ZS)_ proposals lead to an unfeasible low acceptance rate in this case, standard DREAM_(ZS)_ proposals are employed.

### Results

**Fig. 4:**
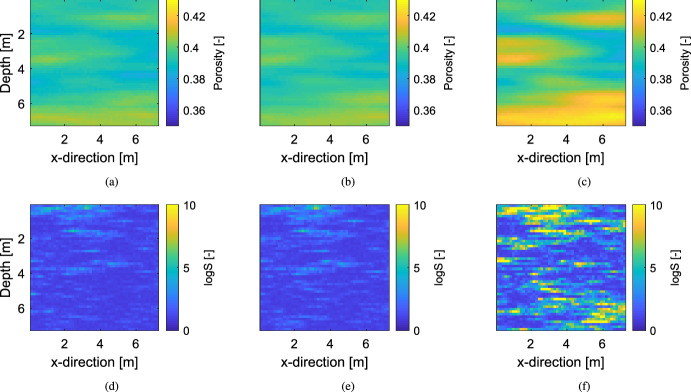
Estimated posterior means of the porosity field $\varvec{\theta }$ obtained with (**a**) the LinGau method, (**b**) the CPM method and (**c**) an inversion ignoring the petrophysical prediction uncertainty. Corresponding pixel-wise logarithmic scores assessing the estimated posterior PDFs for (**d**) the LinGau method, (**e**) the CPM method and (**f**) the inversion ignoring the petrophysical prediction uncertainty

Figure 4a, b display the posterior mean estimates of the LinGau and the CPM method, respectively. Both images look very similar and the structural resemblance to the true porosity field in Fig. 3a is high. Both methods need about 100,000 MCMC iterations to converge and have a very similar acceptance rate (AR) of 10%. They also lead to a similar performance in terms of posterior exploration as both methods sample the true porosity value in all of the pixels and have very similar median standard deviations and logarithmic scores (Fig. 4d, e; Table 1). By comparing the estimated posterior means of LinGau and CPM (Fig. 4a, b) with the one of the inversion ignoring the petrophysical prediction uncertainty (Fig. 4c), it is found that the mean estimate of the inversion ignoring the petrophysical prediction uncertainty has larger amplitudes even if the mean estimates are structurally similar. Its posterior exploration is less extensive, leading to a higher median logarithmic score (Fig. 4f), roughly half the median posterior standard deviation and many pixels that never sample the corresponding true porosity value (about one eighth of the pixels; Tab. 1). For this example exhibiting weak non-linearity and rather small petrophysical prediction uncertainty, it is concluded that the performance for LinGau and CPM are similar. On the other hand, ignoring petrophysical prediction uncertainty leads to biased estimates and too small uncertainty bounds.

**Table 1:** Summary of the results for the Gaussian porosity field example: the acceptance rates (AR), the convergence (Conv) showing the number of iterations needed for the 99th percentile of the parameter’s ${\hat{R}}$-statics to be below 1.2, the percentage of pixels in which the true porosity value $\varvec{\theta } _{\textrm{true}}$ lies within the range of posterior samples, the median logarithmic score (logS) and the median posterior standard deviation (Post SD)

Method	AR (%)	Conv	$\varvec{\theta } _{\textrm{true}}$ (%)	logS	Post SD
LinGau	10	108,000	100.00	1.10	$10.7 \times 10^{-3}$
CPM	10	96,000	100.00	1.16	$10.5 \times 10^{-3}$
No PPE	15	104,000	87.24	2.45	$5.2 \times 10^{-3}$

## Sensitivity Analysis

**Fig. 5:**
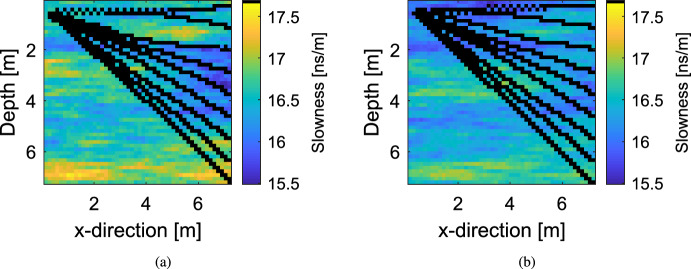
Exemplary GPR ray paths for (**a**) the true slowness field ${\textbf{x}} _{\textrm{true}}$ of the test case presented in Sect. 3 and (**b**) the slowness field resulting from the true porosity field $\varvec{\theta } _{\textrm{true}}$ when ignoring the PPE $\varvec{\varepsilon _{P}}$

In the previous synthetic test case, both the LinGau and the CPM method perform comparably well. As the computational cost of the LinGau method is lower (no need for *N* repeated sampling of the latent variable at each iteration), the use of the LinGau method would be recommended in such a setting. However, the degree of non-linearity in the geophysical forward operator is rather low for this test case. This is illustrated in Fig. 5 showing exemplary ray paths for the true slowness field ${\textbf{x}} _{\textrm{true}}$ (Fig. 5a) and the slowness field based on the true porosity field $\varvec{\theta } _{\textrm{true}}$ but ignoring the PPE (Fig. 5b): most ray paths are close to linear and they are very similar for both fields. Since the LinGau approximation of the likelihood (Eq. 8) relies on a first-order Taylor expansion of the physical forward solver (Eq. 7), it deteriorates when adding PPE $\varvec{\varepsilon _{P}}$ realizations lead to different ray-paths than the field around which the linearization is made. For a linear geophysical relationship, the first-order Taylor expansion is exact for any degree of Gaussian petrophysical prediction uncertainty.

In practice, the question is how to decide, for a given setting, if the LinGau approximation of the likelihood is accurate enough. To shed light on this, a sensitivity analysis exploring the performances of the LinGau and CPM methods for different levels of petrophysical prediction uncertainty is performed. To make the comparison more didactic and to avoid unrelated challenges associated with a very-high dimensional target space, the porosity field is assumed to be layered. Generally, the same setup as in Sect. 3 is considered, but only with 13 transmitters and receivers (169 data points). The observational error is assumed to have a standard deviation of 1 ns.

**Fig. 6:**
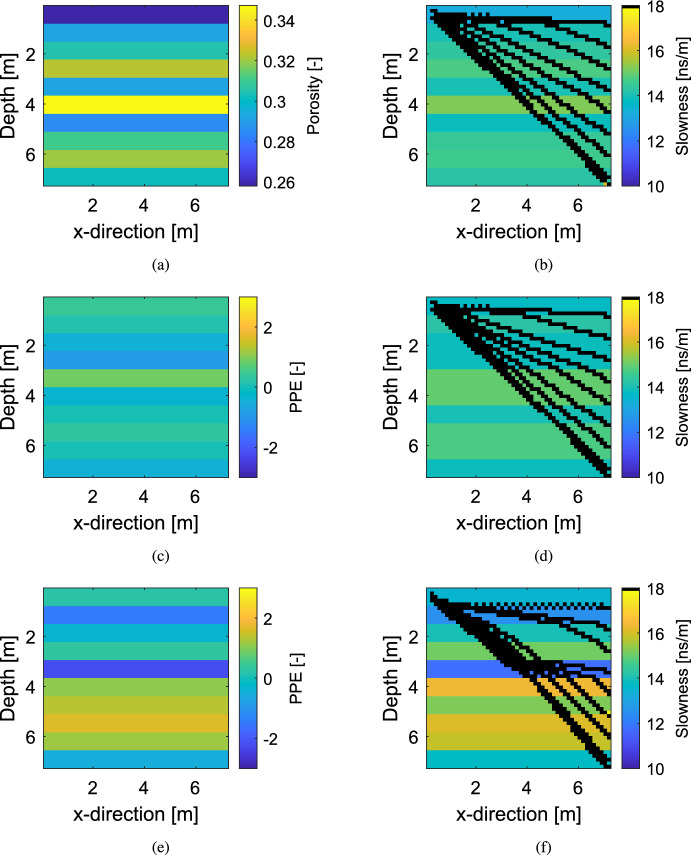
Synthetic “true” models for the layered test cases with the (**a**) same porosity field $\varvec{\theta } _{\textrm{true}}$ and different PPE for (**c**) Setting 1 and (**e**) Setting 2. The right column shows the slowness fields with exemplary ray paths for (**b**) the true porosity $\varvec{\theta } _{\textrm{true}}$ without adding PPE, (**d**) Setting 1 and (**f**) Setting 2

**Fig. 7:**
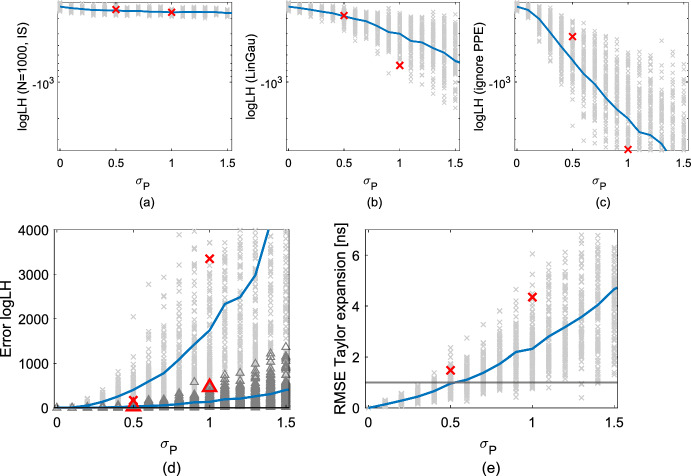
Estimators of log-likelihood $p({\textbf{y}} | \varvec{\theta } _{\textrm{true}})$ of the true porosity field in the layered setting as a function of petrophysical prediction uncertainty ($\sigma _{P}$): (**a**) importance sampling estimate using 1000 samples of the PPE, (**b**) LinGau estimate and (**c**) estimate ignoring the PPE. For each value of $\sigma _{P}$, the same underlying porosity field $\varvec{\theta } _{\textrm{true}}$ is used with 100 different realizations of the PPE, leading to 100 data sets each. The crosses indicate the values for each data set and the solid lines their mean. (**d**) The mean absolute difference between the values of (**a**) and (**b**) are shown in darkgrey triangles and (**a**) and (**c**) in lightgrey crosses. (**e**) The RMSEs in the first-order Taylor expansion (Eq. 7) of the data sets; the black horizontal line shows the standard deviation of the observational noise (1 ns). The red symbols in (**a**)–(**e**) indicate the errors of the settings used in the subsequent inversion examples; thereby in (**d**), the red triangles refer to the errors obtained when using LinGau and the red crosses refer to the errors obtained when ignoring PPE

### Likelihood Estimation

The “true” porosity field $\varvec{\theta } _{\textrm{true}} = (\theta _1,\ldots ,\theta _{10})$ is generated by assuming 10 horizontal layers of equal thickness and drawing independently from a Gaussian distribution with mean 0.3 and standard deviation 0.03 (Fig. 6a). The resulting slowness field (Eq. 11) is distorted with a layered PPE field having zero mean and independent layers with standard deviation $\sigma _{P}$. For each of sixteen different standard deviation values $\sigma _{P}$ ranging from 0.0 to 1.5, one hundred data sets are generated using the same porosity field $\varvec{\theta } _{\textrm{true}}$ (Fig. 6a) but different realizations of the PPE $\varvec{\varepsilon _{P}}$. Thereby, the true log-likelihood value $p({\textbf{y}} | \varvec{\theta } _{\textrm{true}})$ of $\varvec{\theta } _{\textrm{true}}$ has a different value depending on the realization of the PPE and the observational noise, even for the same $\sigma _{P}$. Two exemplary PPE fields and resulting slowness fields with $\sigma _{P}=0.5$ and $\sigma _{P}=1.0$ are depicted in Fig. 6c–f, respectively.

For each of the one hundred data sets per value of $\sigma _P$, $p({\textbf{y}} | \varvec{\theta } _{\textrm{true}})$ is approximated by using the LinGau approach. The corresponding values and their mean for the different $\sigma _P$ are shown in Fig. 7b. Those values are compared with the log-likelihood estimates obtained under the assumption of no PPE (Fig. 7c). Eventually, the aim is to assess these estimates by comparing them to the true log-likelihood values $p({\textbf{y}} | \varvec{\theta } _{\textrm{true}})$ of the corresponding data set. Due to the intractability inherited by the latent variable model, the analytical solution remains elusive and the unbiased importance sampling estimate of Eq. 9 employing 1000 realizations of the PPE (Fig. 7a) is applied. Due to the high number of samples, a well-specified importance density and the low dimensionality of the problem at hand, this estimator is close to the true log-likelihood value. To show that this assumption is valid, the standard deviation of the importance sampling estimator of $p({\textbf{y}} | \varvec{\theta } _{\textrm{true}})$ for both the exemplary settings with $\sigma _{P}=0.5$ and $\sigma _{P}=1.0$ of Fig. 6 is evaluated (indicated with red symbols in Fig. 7). For both $\sigma _{P}=0.5$ and $\sigma _{P}=1.0$, the standard deviation is below one for log-likelihood values around -260. With the underlying assumption of the estimators in Fig. 7a being close to the true value, they are compared with the ones of the LinGau method (Fig. 7d, darkgrey triangles). The absolute errors in the log-likelihood estimation of LinGau increase with increasing $\sigma _P$. While the mean absolute error in the log-likelihood estimator for $\sigma _P=0.5$ is around 30, it grows to 150 for $\sigma _P=1.0$ and to 400 for $\sigma _P=1.5$. However, in comparison to the errors of the method ignoring the PPE (Fig. 7d, lightgrey crosses), the errors of the LinGau approach are comparably small. When ignoring the PPE completely, the mean absolute error for $\sigma _P=0.5$ is about 400, for $\sigma _P=1.0$ it is 1700 and for $\sigma _P=1.5$ even 5000. Thereby, the method ignoring the PPE almost always underestimates the true log likelihood values $p({\textbf{y}} | \varvec{\theta } _{\textrm{true}})$ as it cannot account for the true PPE and therefore gives reduced likelihood to the true porosity field $\varvec{\theta } _{\textrm{true}}$. The same holds true for the LinGau approach with increasing $\sigma _P$ as the method accounts for an increasingly wrong PPE.

The LinGau approximation relies on a first-order Taylor expansion of the physical forward solver (Eq. 7). This approximation deteriorates with increasing degree of PPE as can be seen in Fig. 7e depicting the root mean square errors (RMSE) of the Taylor expansions for the data sets with increasing $\sigma _P$. While the mean of the RMSEs is comparable to the observational noise for $\sigma _P=0.5$, it is twice as large for $\sigma _P=1.0$. To establish the influence of the discussed errors on the inversion results, the setups introduced in Fig. 6 that are indicated with red symbols in Fig. 7 are considered: The first with $\sigma _{P}=0.5$ employs rather small errors, however, the standard deviation of the PPE is twice as high as in the case-study in Sect. 3. In the second setting with $\sigma _{P}=1.0$ the errors are doubled and a realization is targeted where the error in the LinGau likelihood approximation is especially high (about 460 while the mean error for $\sigma _{P}=1.0$ is 150).

### Inversion

Using the two layered synthetic data sets (Fig. 6), the MH algorithms are run with three chains in parallel. Due to the simplicity of the problem, a basic Gaussian random walk is used as proposal scheme, within which, for comparison purposes, a step width (standard deviation) of 0.005 is applied for all methods. Furthermore, the linearizations used in the LinGau and CPM methods are updated at every MCMC iteration to prevent any errors resulting from less frequent updates. For the CPM method, importance sampling and a configuration of $N=3, \rho =0.9$ for the first and $N=50, \rho =0.975$ for the second setting is used. For the layers of the porosity field, independent Gaussian prior PDFs with mean 0.3 and standard deviation 0.03 and for the PPE layers, independent centred Gaussian priors with a standard deviation of 0.5 and 1.0 are assumed, respectively. Finally, for the likelihoods uncorrelated Gaussian observational noise with a standard deviation of 1 ns is assumed.

**Fig. 8:**
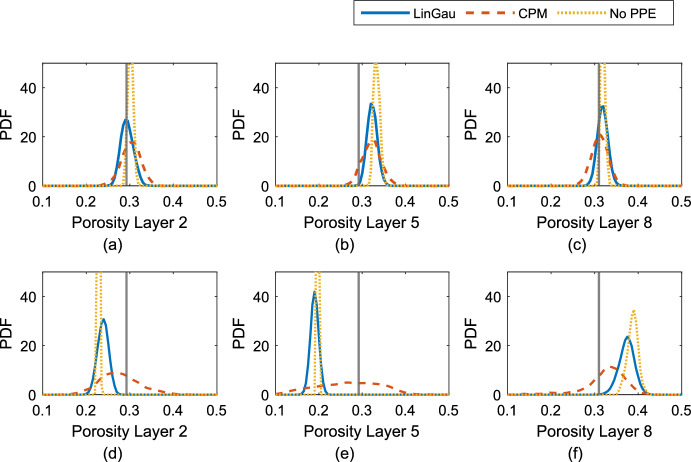
Estimates of the marginal posterior PDFs for the layered test cases: Setting 1 for (**a**) layer 2, (**b**) layer 5 and (**c**) layer 8. Setting 2 for (**d**) layer 2, (**e**) layer 5 and (**f**) layer 8. The solid vertical lines indicate the true porosity values

The estimated marginal posterior PDFs for both settings are shown in Fig. 8 for three of the ten layers. These results are representative of the other layers, but a summary of the performance for all layers is for completeness provided in Table 2. For the first setting (Fig. 8a–c), the modes of all considered methods are located around rather similar values. While the method ignoring the PPE generates samples that do not include the true value of the porosity in half of the layers, both LinGau and CPM capture the true value in all of the ten layers (Table 2). However, while the LinGau method already doubles the standard deviation of the posterior samples compared to the method ignoring the PPE, the CPM method leads to a further doubling compared to LinGau (Table 2). Compared to the method ignoring the PPE, the LinGau method reduces the median logarithmic score from 3.53 to $-$2.37 and the CPM method reduces it even further to $-$2.71. For the second setting (Fig. 8d–f), rather different posterior estimates are obtained for the three methods. The method ignoring the PPE generates posterior samples which are far from the true porosity values and with a small standard deviation (Table 2). The realizations obtained with the LinGau method have a twice as high standard deviation but are also centered far from the true values. The CPM method yields posteriors three times wider than those obtained with LinGau. Thereby, while the posterior samples obtained with the CPM method include the true values of the porosity in all layers, the LinGau method misses them for two layers and the method ignoring the PPE in seven layers. The median logarithmic score of the CPM method ($-$2.00) is distinctly lower than the one of the LinGau method ($-$1.35), which in turn is dramatically lower than for the method ignoring the PPE (378.77).

**Table 2:** Summary of the results obtained for the study targeting a layered porosity field: the acceptance rates (AR), convergence with respect to the ${\hat{R}}$-statics (Conv), the number of layers in which the true porosity value lies within the range of posterior samples ($\varvec{\theta } _{\textrm{true}}$), the median logarithmic score (logS) and the median posterior standard deviation (Post SD)

Method	Setting	AR (%)	Conv	$\varvec{\theta } _{\textrm{true}}$	logS	Post SD
LinGau		40	2500	10/10	$-$2.37	$11.8 \times 10^{-3}$
CPM	1 ($\sigma _{P} = 0.5$)	40	12,000	10/10	$-$2.71	$21.9 \times 10^{-3}$
No PPE		2	13,000	5/10	3.53	$4.2 \times 10^{-3}$
LinGau		40	6000	8/10	$-$1.35	$15.0 \times 10^{-3}$
CPM	2 ($\sigma _{P} = 1.0$)	30	51,000	10/10	$-$2.00	46.4 $\times 10^{-3}$
No PPE		2	20,000	3/10	378.77	$7.2 \times 10^{-3}$

## Discussion

In this work, non-linear geophysical inversion problems involving uncertain petrophysical relationships are targeted. Two different approaches to account for the corresponding intractable likelihood function are explored: a linearized Gaussian approximation (LinGau) and the correlated pseudo-marginal (CPM) method. The performance of these two methods for examples with increasing petrophysical prediction uncertainty is assessed. This work is a continuation of Friedli et al. (2022) as it is the first time the LinGau method is employed and compared with the CPM method. A synthetic crosshole travel-time tomography is first considered, with the aim of inferring a water-saturated multivariate Gaussian porosity field in a situation of only moderate non-linearity and petrophysical prediction uncertainty (adapted from Friedli et al. 2022). The results obtained with the LinGau and the CPM methods are very similar (Fig. 4; Table 1), thereby, indicating that the approximate LinGau method works well in this setting. The LinGau method is then to be preferred as it only necessitates one forward simulation for each MCMC chain and iteration, while CPM with $N=10$ (number of latent variable samples) uses ten times as many forward simulations. If parallelization with $N=10$ more processors are employed, then the computational time can still be made similar to the LinGau approach even if the overall computational cost is significantly higher. For this test case, one iteration for one MCMC chain on a standard laptop takes about 0.3 s for LinGau and 2.1 s for CPM ($N=10$ and no parallelization). Importantly, this example demonstrated that an inversion that ignores petrophysical prediction uncertainty (the most common case in the literature) leads to biased estimates and underestimations of the widths of the posterior estimates by a factor of two.

In Sect. 4, the effect of the degree of petrophysical prediction uncertainty on the likelihood estimation and inversion results is studied for the different methods. To simplify the comparison, layered porosity and PPE fields are used. By comparing log-likelihood estimates, it is shown that the errors in the LinGau method increase with increasing degree of petrophysical prediction uncertainty, even if these errors are much smaller than for the method ignoring petrophysical prediction uncertainty (Fig. 7). By selecting one data set with twice as high petrophysical prediction uncertainty as in the previous multivariate Gaussian example ($\sigma _{P}=0.5$, Fig. 6c) and one with four times as high uncertainty ($\sigma _{P}=1.0$, Fig. 6e), the influence of the likelihood estimation errors on the inversion results is investigated. For the first setting (moderate degree of PPE), the LinGau method performs less well than CPM (underestimation of posterior uncertainty by a factor of two; higher logS scores). However, employing the LinGau method still enables a reasonable approximation of the posterior modes of the layers. Although the true log-likelihood value $p({\textbf{y}} | \varvec{\theta } _{\textrm{true}})$ is underestimated by about 30 with LinGau (Fig. 7d), the RMSE of the Taylor approximation is roughly on the same order of magnitude as the observational error (Fig. 7e). That is different in the second setting with a higher degree of PPE, where the true log-likelihood value $p({\textbf{y}} | \varvec{\theta } _{\textrm{true}})$ is underestimated by about 460 and the RMSE of the Taylor approximation is four times as high as the observational noise. The resulting deterioration in performance for the LinGau method is drastic: underestimation of the posterior uncertainty by a factor of three and twice as high difference in the logS score to CPM than for the first setting. The growing error in the LinGau estimate is due to the Jacobian ${\textbf{J}}_{{\mathcal {F}}(\varvec{\theta } )}$ being increasingly different when considering or not considering the PPE (Fig. 6b, f). For the CPM method, the growing petrophysical prediction uncertainty is accompanied by a very important increase in the posterior standard deviation of the samples (Fig. 8d–f), guaranteeing that the method samples the true porosity values even if the mode of the posterior may be located at the wrong place. Even if the LinGau method still provides much better results than the common approach of ignoring PPE altogether, the linearization on which this method is based (Eq. 7) is unable to account for non-linear effects associated with specific PPE realizations. In contrast, the CPM method only relies on the linearization to derive an importance sampling distribution and errors in this distribution will only lead to slower convergence of the CPM method while still targeting the true posterior distribution.

The approximation error of the first-order Taylor expansion used in the LinGau method (Eq. 7) grows with increasing non-linear effects related to the PPE. By comparing the forward operators for latent variables with (${\mathcal {F}}(\varvec{\theta } ) + \varvec{\varepsilon _{P}}$) and without considering the PPE $\varvec{\varepsilon _{P}}$ (${\mathcal {F}}(\varvec{\theta } )$), this relation can be established (as in Figs. 5 and 6). It is seen that high standard deviations in the $\varvec{\varepsilon _{P}}$ have an adverse effect on the approximation accuracy by highly influencing the forward operator. If the PPE $\varvec{\varepsilon _{P}}$ strongly influences the Jacobian (as in Fig. 6e), caution is advised when applying the LinGau method (Fig. 7d). To choose between the LinGau and the CPM method, this study recommends to investigate the RMSE of the first-order Taylor expansion (Fig. 7e) and to compare it with the observational noise. For a setting when the RMSE is significantly lower than the observational error (as for $\sigma _P=0.25$ in Sect. 3), the recommendation is to use the LinGau method due to its lower computational cost (the effect of the petrophysical prediction uncertainty is incorporated into the likelihood function and no importance sampling of latent variables is needed). If the RMSE is in the same order of magnitude as the observational error (as for $\sigma _P=0.5$ in Sect. 4), the LinGau method can be applied if moderate errors in the posterior estimation are acceptable. If the RMSE is clearly higher than the observational error (as for $\sigma _P=1.5$ in Sect. 4), the exact CPM method should be used. If this is computationally too expensive, one could consider accounting for the approximation error in the LinGau method by treating it as a model error term (see e.g. Hansen et al. 2014).

The approximation underlying the LinGau approach could easily be incorporated in deterministic inversion methods (using gradients), while this is impossible for CPM. On the other hand, while the LinGau method requires Gaussian assumptions for the PPE, the CPM method can sample the latent variables from a variety of possible distributions. But in practice the CPM method needs a well-working importance sampling strategy to be efficient. For the presented test cases, a decreasing acceptance rate for the CPM method is observed with increasing petrophysical prediction uncertainty if the number of latent variables *N* and correlation $\rho$ are fixed. This occurs as our importance sampling scheme gets more and more inaccurate, which can be compensated by using larger *N* or $\rho$. Although the efficiency is reduced, the accuracy of the posterior samples remains the same as the importance sampling is only used to decrease the variance of the likelihood estimator. In contrast, the LinGau method does not only loose efficiency with increasing non-linearity, but also leads to overconfident and biased estimates of the target parameters as the approximation of the likelihood is getting increasingly inaccurate (Fig. 8).

In the presented test cases, the mean and the covariance structure of the target and petrophysical prediction error fields are assumed to be known. In a setting where they are unknown, hierarchical Bayes could be employed (as e.g. in Laloy et al. 2015; Brunetti and Linde 2017). A recent study by Friedli et al. (2023) targets a setting where the problem is formulated differently: only the posterior mean and the covariance structure are derived, while the small-scale variations in the model domain are accounted for but not inferred. In this study, a crosshole ray-based setting that is only weakly non-linear is considered. More non-linear problems such as electrical resistivity tomography or surface-based seismic refraction tomography might exhibit an even stronger sensitivity to petrophysical prediction uncertainty and the applicability of the LinGau might be reduced compared with the present study. Indeed, the errors in the LinGau approximation are not due to the petrophysical predication uncertainty as such, but rather how individual realizations of it affects the sensitivity patterns compared to the Taylor expansion that is performed in absence of petrophysical prediction uncertainty. Even if the focus herein is on how to account for petrophysical prediction uncertainty, both the LinGau and the CPM methods could be adapted to, for instance, account for 3-D effects in 2-D inversions, variations in porosity in tracer-test tomography targeting the permeability field, or to account for hydraulic storativity fluctuations in hydraulic tomography studies. Indeed, this type of latent variable problem arises as soon as the measured data do not only depend on the main parameters of interest but also on some other variables influencing the response.

## Conclusions

This work focus on geophysical inversion problems targeting the posterior distribution of (hydro)geological parameters while accounting for uncertain petrophysical relationships and non-linear physics. The resulting intractable likelihood function is accounted for by either the linearized Gaussian approximation (LinGau) method or the correlated pseudo-marginal (CPM) method. The LinGau method, so far untested in geophysical inversion, is an approximate method that is computationally cheaper than CPM as it does not necessitate a Monte Carlo estimation of the likelihood at each MCMC iteration. In a first case study, a water-saturated multivariate Gaussian porosity field is considered for which the scatter in the petrophysical relationship and the non-linearity is comparatively small. In this setting involving crosshole first-arrival travel times, the LinGau method succeeds equally well as CPM in exploring the posterior distribution. For comparison, an inversion ignoring petrophysical uncertainty provides biased results and too narrow uncertainty estimates. In a subsequent sensitivity analysis considering layered fields, the degree of petrophysical prediction uncertainty is increased, thereby introducing increasing inaccuracies associated with the Taylor expansion on which the LinGau method is based. Consequently, the LinGau method produces increasingly inaccurate results as the petrophysical prediction uncertainty grows such that the true values are more and more often unsampled and the logarithmic scores are high. In contrast, the CPM method performs very well for all settings and accommodate the growing uncertainty in the petrophysical uncertainty, while this is only partially achieved by the LinGau method. The computationally less intensive LinGau method is attractive when the impact of the scatter of petrophysical prediction uncertainty is small compared to the observational noise. In comparison, the computationally more costly CPM method is an exact and much more general method that clearly outperforms the LinGau method when the petrophysical uncertainty grows in magnitude, but it needs an efficient importance sampling distribution to work well in practice. If the CPM method is computationally too expensive for a given application and if petrophysical uncertainty is significant, it is still better to use the LinGau method than inversions ignoring petrophysical prediction errors as the resulting results are less biased and the underestimation of posterior uncertainty is less pronounced.
